# Acquired neuromyotonia following upper respiratory tract infection: a case report

**DOI:** 10.4076/1757-1626-2-7952

**Published:** 2009-09-08

**Authors:** Ibrahim Imam, Simon Edwards, C Oliver Hanemann

**Affiliations:** 1 Neurology Department, Torbay HospitalTorquay TQ2 7AAUK; 2 Neurology Department, Derriford HospitalDerriford Road, Plymouth PL6 8DHUK; 3 Peninsula College of Medicine and DentistryJohn Bull Building, Tamar Science Park, Research Way, Plymouth PL6 8BUUK

## Abstract

We present a 37-year-old male subject who presented with burning sensations in his hands and feet with generalised twitching of his limbs, trunk and face. His symptoms developed 2 weeks after an upper respiratory tract infection. There was associated facial flushing and disturbed night sleep but no memory impairment or generalised sweating. Examination showed generalised myokymia and fasciculations and electromyography revealed widespread continuous semi-rhythmic doublets and triplets of low frequency with interspersed silent periods. Anti voltage gated potassium channel antibodies, antinuclear antibodies, anti-acetylcholine receptor antibodies and the anti-neuronal antibodies anti Hu, anti Yo and anti Ri were all negative. His symptoms improved slightly on lamotrigine and amitriptyline.

## Introduction

Neuromyotonia is a form of peripheral nerve hyperexcitability with spontaneous and continuous muscle fibre activity [1]. It has variously been called undulating myokymia, Isaac’s syndrome and cramp-fasciculation syndrome [2]. It might be hereditary or acquired and there have been a variety of reported causes and associations. Only three cases have been however been attributed to infective causes in the literature. We present a case of acquired neuromyotonia following an upper respiratory tract infection and discuss the literature relevant to the case.

## Case presentation

Our patient is a 37-year-old Caucasian man who presented with a fourteen-month history of a burning, painful sensation in his hands and feet. This developed about two weeks after an upper respiratory tract infection. His symptoms were present all the time but were exacerbated by activity and stress. There was associated twitching of his hands and face as well as regular facial flushing and disturbed night sleep. He had no memory impairment or sweating disturbances. He has a past history of mild renal dysfunction and is on antibiotics for a persistent cellulitis over his left elbow. He does not smoke and takes only minimal alcohol.

On examination, he had generalised myokymia and fasciculations involving all parts of his face, tongue, trunk and all four limbs. Power, coordination and reflexes were normal. He had impaired pain sensation to his ankles and wrists; other modalities of sensation were normal.

Electromyography (EMG) revealed widespread continuous semi-rhythmic doublets and triplets of low frequency with interspersed silent periods. There were fasciculations in the right tibialis anterior and right opponens pollicis with some polyphasia in the tibialis anterior muscle. Motor and sensory nerve conduction velocities were normal. He tested negative for anti voltage gated potassium channel (VGKC) antibodies, antinuclear antibodies and anti-acetylcholine receptor antibodies. He was also negative for the anti-neuronal antibodies anti Hu, anti Yo and anti Ri. His creatinine kinase was mildly elevated at 474 U/L as was serum creatinine at 111-micromol/L. eGFR was mildly reduced at 67. Computerised tomography scan of his chest was normal.

He was initially treated with gabapentin with no benefit. He had improvement in pain and sleep disturbance on 90 mg daily dose of amitriptyline. He also reported further improvement in sensory symptoms on Lamotrigine but not in motor symptoms.

## Discussion

Our patient had most of the typical features of neuromyotonia. The major manifestations are cramps, muscle twitching or stiffness, pseudomyotonia, paraesthesias, numbness and hyperhydrosis [2]. The commonest feature is muscle twitching which is present in over 90% of cases; facial twitching is seen in a quarter of cases [1]. Our patient had both limb and facial involvement. Central nervous system features are seen in a quarter of cases of neuromyotonia and these include mood change, sleep disorders, hallucinations and personality changes [2]. Our patient however did not report central nervous system symptoms.

The only risk factor for neuromyotonia in our patient appears to be the upper respiratory tract infection he had two weeks prior to onset of symptoms. The elbow cellulitis is unlikely to be responsible because there was no temporal relationship to the onset of Neuromyotonia. There have been case reports of neuromyotonia following *Staphylococcus aureus* spinal epidural abscess with septicaemia [3,4], and there is also a report of ocular neuromyotonia following cavernous sinus thrombosis due to mucormycosis [5]. In one of these cases, antibodies to voltage-gated potassium channels were demonstrated during the infection, and these disappeared after resolution of the infection [3]. Although these antibodies were negative in our patient, they were only tested for after he had recovered from the respiratory tract infection. It is therefore possible that similar antibodies underlie the pathogenesis in our patient and other cases resulting from infective causes.

Infections are however uncommon reported causes of neuromyotonia. More frequently reported are associations with autoimmune diseases including myasthenia gravis, systemic lupus erythematosus, dermatomyositis and Hashimoto’s disease. Neuromyotonia may follow bone marrow transplantation, radiation therapy as well as wasp stings. It has also been reported on the background of both acquired demyelinating [6], as well as hereditary, motor neuropathy [7]. Associations with malignancies are with thymoma, Hodgkin’s lymphoma and renal cell carcinoma. Neuromyotonia is seen in anti VGKC antibody limbic encephalitis but in only 10% of patients [8].

Our patient had the characteristic EMG features of neuromyotonia and these are spontaneous motor unit discharges, which are thought to originate from the distal parts of the nerves. Typical findings are myokymic doublet or multiplet motor discharges, the former being the more common [2]. There is however no correlation between abnormal findings and clinical severity of involved muscles [2]. Subjects with abnormal EMG findings are reportedly more likely to have anti VGKC antibodies than those with normal EMG findings, and these antibodies are seen in about 40% of cases of neuromyotonia [2]. Patients with abnormal EMG features are also more likely to have autoimmune diseases like myasthenia gravis and are more likely to report significant disability [2]. Our patient had elevated creatinine kinase levels and this has been reported in half of cases of neuromyotonia [2].

The major differential diagnoses of neuromyotonia are stiff man syndrome (in which the discharges disappear in sleep) and motor neurone disease, in which neuromyotonic discharges can be seen early on in the course [1]. Our patient does not appear to have any of the above features.

Treatment for neuromyotonia is with anticonvulsant agents. Phenytoin, carbamazepine, sodium valproate and lamotrigine may be used singly or in combination [1]. It is recommended that immunosuppression with prednisolone and azathioprine, or plasma exchange be considered if these are unsuccessful [9,10]. Immunoglobulin therapy however seems not to be helpful [1]. Our patient’s sensory symptoms had improved with symptomatic treatment with amitriptyline. Lamotrigine also improved his painful symptoms but has so far not reported improvement in muscle twitching.

## Abbreviations

eGFR: estimated glomerular filtration rate
EMG: electromyography
VGKC: voltage gated calcium channel
